# Optimization and Randomized Controlled Evaluation of Plantar White Noise Vibration for Balance Improvement in Young Adults

**DOI:** 10.3390/s26092709

**Published:** 2026-04-27

**Authors:** Zhiyu Wu, Jinkun Xie, Chunlian Xi, Xiaobo Song, Bingshan Hu

**Affiliations:** 1 School of Health Science and Engineering, University of Shanghai for Science and Technology, 516 JunGong Road, Shanghai 200093, China; zhiyu_wu@163.com (Z.W.); 18567285086@163.com (J.X.); 2 Department of Rehabilitation, Shidong Hospital, Yangpu District, Shanghai 200438, China; 15109607197@163.com; 3 School of Physical Education and Sports, Central China Normal University, Wuhan 430079, China; songxb@ccnu.edu.cn

**Keywords:** postural sway, stochastic resonance, electromyography, sensorimotor training

## Abstract

**Highlights:**

**What are the main findings?**

- Threshold-level plantar white-noise vibration combined with multi-site plantar stimulation may help improve postural control in healthy young adults.
- The intervention showed no marked change in ankle muscle activation, suggesting that the observed balance-related effects may be associated with sensory modulation rather than direct motor enhancement.

**What are the implications of the main findings?**

- The 8-week plantar vibration intervention was associated with a reduction in the center-of-pressure envelope area during single-leg stance, suggesting a potential sustained effect on balance performance.
- These findings may provide a basis for further optimizing plantar vibration protocols for balance rehabilitation and injury prevention.

**Abstract:**

Postural control is essential for daily function, and while stochastic resonance (SR) enhances balance in clinical populations, its efficacy in healthy young people remains underexplored. This study investigated (1) biomechanical effects of multisite plantar vibration on postural stability using center-of-pressure (CoP) parameters, and (2) short-term and sustained effects on balance performances. Phase 1 enrolled six participants to identify the optimal plantar stimulation configuration and to evaluate acute electromyographic responses under threshold-level vibration. Phase 2 evaluated long-term efficacy through an eight-week sham-controlled parallel-group randomized controlled trial. In this trial, eight participants received vibration combined with balance training, and another eight participants completed the same training protocol using sham insoles without vibration, analyzing CoP parameters (95% ellipse area, path length) and muscle activation (tibialis anterior, medial gastrocnemius, peroneus longus, extensor digitorum longus). Results showed full-site vibration reduced CoP area versus control (265.66 ± 188.6 mm^2^ vs. 437.84 ± 190.95 mm^2^, *p* < 0.05) without altering ankle muscle activation (all *p* > 0.05). Longitudinal analysis revealed CoP area reduction (−4.88 ± 10.42%) in the intervention group versus sham (*p* < 0.001), with maximum anterior displacement increasing by 25.03% during vibration (*p* < 0.05). Plantar white-noise vibration modulates CoP oscillations without neuromuscular activation changes, demonstrating that full-site stimulation acutely enhances postural stability while sustained intervention improves dynamic balance control.

## 1. Introduction

Postural control, essential for maintaining body position during daily activities, relies on integrated sensory feedback from three primary systems: the visual system, which provides spatial orientation through retinal slip [1,2,3,4]; the vestibular system, which detects linear and angular head motion relative to gravity and inertia [5]; and the somatosensory system, which integrates signals from cutaneous, muscular, and articular receptors to convey positional and kinematic information [6]. The central nervous system synthesizes these multisensory inputs to estimate body position in space and generates appropriate motor commands to maintain postural stability [7,8]. A key aspect of this process is the counteraction of destabilizing torque from external forces by corrective torque arising from foot–ground interactions, wherein the plantar surface dynamically responds to ground reaction forces [9,10]. Moreover, the foot integrates proprioceptive inputs from ankle joint receptors [11], muscle spindles, and cutaneous exteroceptors, establishing itself as the primary sensorimotor interface for upright postural control [12,13].

Stochastic resonance theory offers an innovative approach to enhancing sensorimotor function. As a neuromodulation method, stochastic resonance applies random stimulation at specific subliminal or supraliminal intensities to sensory systems and has been shown to improve human balance performance [14,15]. This enhancing effect extends across multiple sensory modalities, including tactile, auditory, and visual systems [16]. The human body functions as a nonlinear dynamic system capable of utilizing white noise signals with a constant power spectral density as an ideal source of subliminal stimulation [17,18,19]. As described by Collins et al. [20], the core mechanism involves superimposing Gaussian white noise at an optimal intensity onto intrinsic neural activity, which allows subthreshold signal fluctuations to transiently exceed the activation threshold of sensory receptors, thereby facilitating detection by the central nervous system. Empirical evidence confirms that white noise signals reduce tactile perception thresholds [21], thereby enhancing sensory sensitivity. Building on this principle, research by Cham et al. [22] demonstrates that subliminal tactile vibration delivered via insoles lowers sensory thresholds and improves the sensitivity of plantar mechanoreceptors, optimizing somatosensory feedback for postural adjustment and thereby enhancing balance control in older adults [23,24].

The effectiveness of such interventions rests on a critical physiological foundation. Plantar skin is densely populated with mechanoreceptors—approximately 100 per foot [25]—that exhibit high sensitivity to vibrational and stretch stimuli [26,27] and contribute to fine-tuning the center of pressure within the base of support [28]. Accurate plantar tactile information is essential for maintaining both static and dynamic balance [29]. Conversely, impairing plantar input through methods such as foot cooling [30], local anesthesia [31], or vibration-induced muscle fatigue [32] compromises postural control, a process largely mediated by the muscle spindle reflex pathway [33,34]. It should be noted, however, that because healthy young adults already possess efficient and intact sensorimotor integration, stochastic resonance interventions in this population often produce only limited observable improvements in overall behavioral outcomes such as postural sway [15,23]. This suggests that any neuromuscular effects may be subtle and require more precise measurement techniques, such as electromyography of specific muscles, to be adequately captured.

Evidence suggests that plantar tactile interventions can improve balance in individuals with stroke [15]. Existing studies have largely focused on older adults, with insole-based white noise vibration as the primary intervention [18]. Regarding the effects of plantar vibration stimulation on balance control in young adults, previous studies have reported findings that remain inconsistent. Galica et al. [35] applied subthreshold plantar vibration to healthy young adults and found no significant changes in gait velocity or gait variability measures. Chien et al. [36] reported that 250 Hz plantar vibration produced stronger long-range correlations in stride length compared to 30 Hz and 150 Hz stimulation, suggesting that vibration frequency modulates gait control. Yamashita et al. [37] applied 40–600 Hz suprathreshold plantar vibration to healthy young males and observed a significant reduction in foot trajectory variability during the swing phase. Pathak et al. [38] found that suprathreshold plantar vibration significantly reduced minimum toe clearance variability, whereas subthreshold vibration produced no significant effects. Song et al. [39] investigated obstacle crossing tasks and found that both subthreshold and suprathreshold plantar vibration significantly increased toe clearance height.

The physiological limits of sensorimotor integration in healthy populations provide a baseline against which deficits in pathological conditions can be compared [40]. Balance improvements in healthy individuals are often subtle, which renders stochastic resonance effects subject to a more rigorous test, as ceiling effects can easily mask intervention outcomes [24]. The proportion of young adults engaged in high-intensity physical activities is increasing, and even modest gains in balance could translate into improved athletic performance, particularly among elite athletes, while also contributing to a reduced risk of sports-related injuries [11,41]. Conducting such studies in healthy young adults holds distinct scientific value [23]; however, systematic research in this population remains limited, particularly regarding optimization of stimulation parameters, dose-response relationships, and long-term retention effects [42].

Although evidence indicates that plantar tactile interventions can improve balance in clinical populations such as stroke patients, most existing research has focused on older adults receiving continuous insole micro-vibration. Several key gaps remain in the current literature. First, there is a lack of systematic evaluation regarding how different stimulation sites and parameters influence postural control in healthy young adults. Second, it is unclear whether and how such interventions specifically modulate activation patterns of the major ankle-stabilizing muscles. Third, the persistence of neuromuscular adaptations and postural improvements after cessation of white-noise micro-vibration in healthy young adults requires further investigation. To address these gaps, this study evaluates the immediate effects and after-effect retention of a novel localizable micro-vibration device during a single-leg stance task in healthy young adults. Emphasis is placed on assessing how optimized stimulation sites affect postural control ability and activation patterns of specific muscle groups. We hypothesize that plantar white-noise micro-vibration applied to optimized sites will improve postural stability in healthy young adults, with possible short-term retention after stimulation cessation, while any changes in muscle activation may be limited under static testing conditions.

To address the above gaps, the present investigation was structured into three complementary cohorts with distinct purposes. Cohort 1 served as a preliminary optimization cohort to determine the most effective plantar stimulation sites and parameter configuration, thereby providing the experimental basis for the subsequent intervention study. Cohort 2 was used to evaluate the effectiveness and time-related efficacy of the optimized vibration insole intervention over the 8-week protocol. Cohort 3 was included as the corresponding training control cohort to distinguish the specific effect of the vibration insole from improvements attributable to repeated balance tasks or balance training alone. The third substudy targeted long-term efficacy and was designed as an eight-week sham-controlled parallel-group randomized controlled trial. One cohort received plantar vibration together with balance training, whereas the other cohort completed the same balance training protocol with sham insoles that did not deliver vibration. This design allowed us to isolate the specific contribution of plantar vibration beyond balance training alone.

## 2. Materials and Methods

### 2.1. Experimental Protocol

**Figure 1:**
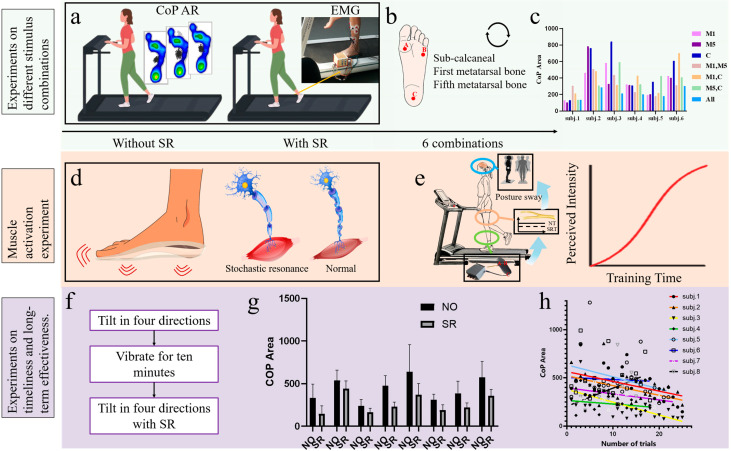
Overview of the experimental design and plantar intervention effects in young adults. (**a**) Plantar pressure and EMG data collection; (**b**) Micro-vibration sites: first/fifth metatarsals and calcaneus; (**c**) CoP envelope area changes across stimulation combinations; (**d**) Subsensory plantar micro-vibration enhances tactile perception; (**e**) Single-leg treadmill stance showing faster postural adjustments with subsensory stimulation; (**f**) Dynamic balance assessment and training protocol; (**g**) Efficacy results; (**h**) Long-term effect results.

The study was structured into three sequential phases with different but complementary objectives. In Phase 1, Cohort 1 was used for preliminary optimization of plantar stimulation sites through systematic testing of three plantar regions (Figure 1b) during balance-related tasks (Figure 1a). The experimental results (Figure 1c) identified the stimulation-site combination that produced the most favorable postural stability response, thereby providing the experimental basis for the design of the micro-vibration insole used in the later phases. In Phase 2, the selected stimulation configuration was used to further determine the optimal vibration intensity and frequency parameters and to investigate the acute effects of plantar vibration on muscle activation levels (Figure 1d). In Phase 3, the optimized stimulation protocol was applied in an eight-week sham-controlled parallel-group randomized controlled trial to evaluate the effectiveness and time-related efficacy of the vibration insole intervention. Sixteen participants aged 24 to 27 years were allocated to two independent cohorts. Cohort 2 received vibration combined with balance training during both training and assessment sessions, whereas Cohort 3 completed the same procedures with the vibration function deactivated using sham insoles. The inclusion of Cohort 3 was intended to exclude improvements attributable solely to repeated balance tasks or balance training, thereby isolating the specific contribution of the vibration insole. This longitudinal design enabled the evaluation of both the immediate and sustained effects of the intervention (Figure 1g,h).

### 2.2. Participants

A total of 22 healthy young adults were recruited and divided into three independent cohorts with distinct but complementary roles in the overall study. Cohort 1, used for parameter optimization, included six participants (3 males, 3 females; mean ± SD: age 23.8 ± 1.3 years, height 167.2 ± 4.1 cm, weight 62.3 ± 10.5 kg, BMI 21.1 ± 0.8). This cohort was enrolled exclusively for preliminary experiments to determine the optimal vibration sites and stimulation parameters, thereby providing the experimental basis for the subsequent intervention study, and did not participate in the later 8-week longitudinal trial. Cohort 2, designated for vibration intervention, included eight participants (6 males, 2 females; mean ± SD: age 23.3 ± 1.5 years, height 167.8 ± 4.8 cm, weight 66.8 ± 13.2 kg, BMI 22.7 ± 3.5). This cohort served as the active intervention arm of the 8-week randomized controlled trial and wore insoles with the optimized vibration function activated during all training and assessment sessions, in order to evaluate the effectiveness and time-related efficacy of the vibration insole intervention. Cohort 3 served as the training control group and included eight participants with demographic characteristics comparable to those of Cohort 2. This cohort completed the same 8-week training and assessment protocol as Cohort 2; however, the insoles worn were identical in appearance but had their vibration function deactivated. The inclusion of Cohort 3 was intended to control for improvements in balance attributable to repeated balance tasks or balance training alone, thereby allowing the specific contribution of the vibration insole to be distinguished.

Participants in the 8-week trial were assigned to groups according to a pre-generated random sequence, and no investigator-directed or manual selection of group assignment was performed. Before testing, all participants received the same explanation of the study purpose and the same standardized task instructions. However, they were not informed of the existence of separate active and sham groups, and they were unaware of their own group allocation.

Inclusion criteria for all participants were age between 18 and 35 years, ability to maintain single-leg stance for at least 13 s, and no history of lower extremity injury within the past 6 months. Exclusion criteria included history of neurological disorders such as stroke, Parkinson’s disease, or peripheral neuropathy; diabetes mellitus; vestibular disorders; lower extremity fracture or surgery within the past year; pregnancy; use of medications affecting balance or proprioception; and participation in balance training programs within the past 3 months. All participants reported no history of stroke, diabetes, or other peripheral neuropathies that could affect plantar sensation. All participants were office workers with a background in academia, primarily graduate students. None had formal training in lower-limb balance exercises or significant sports practice. They had a relatively sedentary lifestyle, with minimal habitual physical activity related to balance training. This study was approved by the Medical Ethics Committee of Shanghai Pudong New Area Gongli Hospital (approval number GLYY1s2024-010). Written informed consent was obtained from all participants.

### 2.3. SR Vibration Device

**Figure 2:**
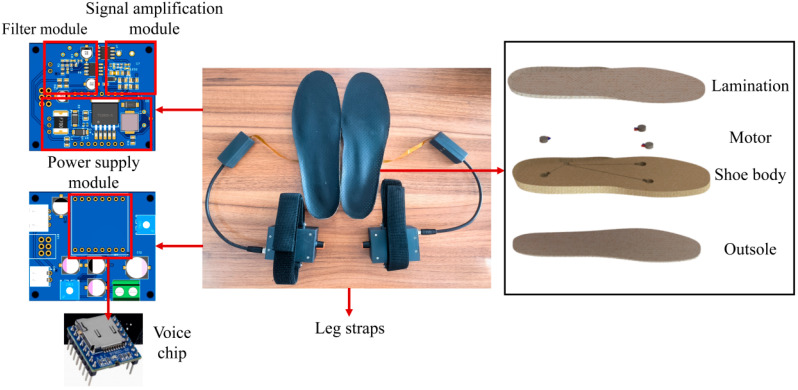
Micro-vibration stimulation insole based on white noise.

This study developed a custom plantar micro-vibration insole for delivering stochastic resonance-based stimulation (Figure 2). The system consists of four integrated hardware modules: a power supply unit, an audio module containing a voice chip that stores prerecorded broadband natural noise, a signal-conditioning unit with a 120 Hz low-pass filter, and a power-amplification stage. During operation, the stored natural noise signal—characterized by a nearly flat spectrum over an effective bandwidth of 20–2000 Hz—is first band-limited by the low-pass filter to concentrate energy within the frequency range to which plantar mechanoreceptors are most sensitive (<120 Hz). The filtered signal is then amplitude-adjusted by the amplifier and delivered to three sets of linear vibration motors, each set embedded beneath a key weight-bearing region of the foot: the first metatarsal (M1), the fifth metatarsal (M5), and the sub-calcaneal (C) area.

Power is supplied by a 12 V rechargeable battery regulated through a dedicated voltage-regulation circuit, with operation controlled by an inline switch. To ensure reliable and unobtrusive wear, 0.3-mm-thick flexible printed circuit (FPC) ribbons connect the amplifier outputs to the motors, replacing conventional wiring to minimize friction and fatigue. All electrical junctions are housed in custom-designed protective enclosures modeled in SolidWorks 2022 (Dassault Systèmes, Vélizy-Villacoublay, France). The motors are seated below a laminated insole surface, and cable outlets are routed through the medial arch to avoid stress and damage during repeated flexion.

### 2.4. Experimental Data Acquisition System

**Figure 3:**
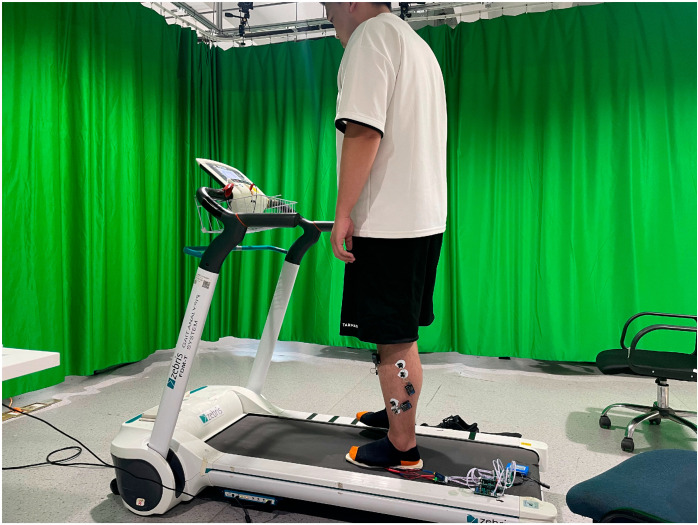
Experimental data acquisition devices.

All experiments were conducted at the Biomechanics Laboratory, School of Health Science and Engineering, University of Shanghai for Science and Technology (Shanghai, China) between September 2024 and February 2025. Testing sessions were scheduled between 9:00 a.m. and 5:00 p.m. to control for circadian variations in balance performance. This experiment synchronized center of pressure and electromyography data acquisition using a Zebris FDM-T pressure platform (Zebris Medical GmbH, Isny, Germany) and ME6000 system (Mega Electronics Ltd., Kuopio, Finland). The Zebris FDM-T pressure platform contained more than 5300 capacitive pressure sensors arranged at a density of 4 sensors/cm^2^, sampling at 120 Hz. System calibration was performed weekly using manufacturer-supplied weight plates. The ME6000 surface electromyography system collected electromyographic signals at a sampling frequency of 2000 Hz with 16-bit resolution, input impedance greater than 10 gigaohms, and a common mode rejection ratio greater than 110 decibels. All EMG analyses reported in this study were based on data acquired using this sampling frequency. Center of pressure parameters including sway area, path length, and velocity were derived within 30 s during standing. Final center of pressure areas were calculated via 95% confidence ellipses fitted to anteroposterior and mediolateral coordinates to assess static postural stability, as illustrated in Figure 3.

Electromyography signals were collected using Ag/AgCl electrodes (Shenfeng Medical Devices, Shanghai, China) with a contact diameter of 10 mm and inter-electrode spacing of 20 mm. Signals underwent hardware band-pass filtering with a frequency range of 10 to 500 Hz and notch filtering from 48 to 52 Hz to eliminate power line interference. Prior to data collection, maximal voluntary contractions were performed to optimize electrode placement on lower limb muscles including the tibialis anterior, medial gastrocnemius, peroneus longus, and extensor digitorum longus, while preventing cable crossover. Post-acquisition MATLAB R2020b (The MathWorks, Natick, MA, USA) scripts computed trial-averaged root mean square values representing mean muscle activation levels.

### 2.5. Effects of Various Combinations of Plantar Neuromodulation on Postural Control Capacity

During human standing, the peak pressure areas of the plantar surface are located at the first metatarsal, fifth metatarsal, and sub-calcaneal. However, the specific enhancement effects of these three regions on balance control remain unclear. In this experiment, the CoP confidence ellipse areas before and after plantar stimulation were measured for each subject. Comparative analysis across experimental groups was conducted to evaluate the impact of different plantar neuromodulation sites on balance control.

**Figure 4:**
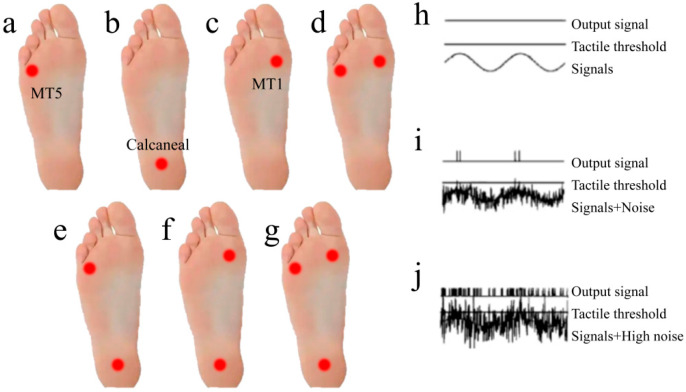
Combinations of stimulation sites and intensities. (**a**) M5; (**b**) C; (**c**) M1; (**d**) M1, M5; (**e**) M5, C; (**f**) M1, C; (**g**) M1, M5, C; (**h**) Low-intensity stimulation; (**i**) White noise stimulation; (**j**) High-intensity stimulation.

The CoP confidence ellipse area was recorded during each stimulation protocol, followed by a 10-min rest period to mitigate potential carryover effects from prior stimuli. Stimulation intensity was individually calibrated based on real-time measurements of each subject’s plantar sensory threshold. Six subjects were selected for this experiment, with all participants undergoing vibratory testing under threshold-intensity conditions. The combinations of stimulation sites and parameter configurations are illustrated in Figure 4.

In investigations of plantar stimulation sites, three peak pressure regions on the plantar surface have been identified, though optimal stimulation parameters for these regions remain undetermined. Previous studies predominantly employed sub-threshold micro-vibration stimuli for plantar interventions, while some research suggests the efficacy of high-intensity stimulation. Consequently, this experimental phase administered plantar vibrations of varying intensities to participants, with optimal stimulation parameters determined through comparative analysis of balance parameters pre- and post-stimulation.

Plantar stimulation studies confirm three peak pressure regions with undefined optimal parameters. Contrasting prevalent sub-threshold micro-vibration applications and emerging high-intensity evidence, this phase administered graded vibration intensities. Optimal parameters were derived from pre-/post-stimulation balance parameter comparisons. Pre-tests measured individual sensory thresholds: stimulus intensity precisely reaching threshold is designated threshold vibration, while suprathreshold intensities are classified as strong vibration.

Balance parameters are recorded under three conditions: no vibration, threshold vibration, and strong vibration. Independent left/right foot assessments mitigate inter-limb variability. All tests occur in soundproof enclosures following 3-min environmental acclimatization. Custom insoles embedding micro-vibration actuators are fixed on a force platform.

Regarding the selection of testing duration, Dingenen et al. [43] employed a 13-s protocol and demonstrated excellent test-retest reliability for CoP displacement, with an intraclass correlation coefficient of 0.91. Hertel et al. [44] used a 10-s duration and successfully detected differences in postural control among different foot types. Based on this evidence, combined with pilot testing in our laboratory confirming the feasibility of healthy young adults maintaining single-leg stance for 13 s, the present study established a single-leg stance testing duration of 13 s. The dominant limb was determined by the average of triplicate non-vibration CoP envelope areas. Participants maintained single-leg stance on the dominant limb for 13 s, ensuring full plantar contact for white-noise delivery to the first metatarsal, fifth metatarsal, and calcaneal regions. Each subject completed three 13-s trials per condition. Unilateral stance amplifies outcomes and reflects gait-cycle postural adjustments.

IBM SPSS Statistics 24 (IBM Corp., Armonk, NY, USA) verifies CoP envelope area normality via Q-Q plots. Paired *t*-tests compare conditions (*p* < 0.05). MATLAB analyzes CoP-vibration parameter correlations.

### 2.6. Effects of Plantar Nerve Stimulation on Muscle Activation

In the study investigating the effects of plantar nerve stimulation on balance control, we identified the first metatarsal, fifth metatarsal, and calcaneus as the optimal stimulation sites, confirming the influence of plantar nerve stimulation on postural stability. To examine the acute effects of combined stimulation at these three sites on balance control and muscle activation, plantar white-noise stimulation was applied to the first metatarsal, fifth metatarsal, and calcaneus. By comparing muscle activation levels before and after vibratory intervention, this study aimed to characterize the effects of plantar white noise stimulation on neuromuscular responses and quantify the correlation between stimulation parameters and changes in muscle activation patterns.

The study recruited six participants. Prior to testing, the vibration insole was placed on the pressure platform, with participants instructed to maintain full contact between their left foot and the insole device. Eight pre-gelled surface-adhesive bipolar electrodes (Ag/AgCl) were attached to the skin over the medial gastrocnemius (MG), tibialis anterior (TA), peroneus longus (PL), and extensor digitorum longus (EDL). To minimize signal cross-talk, electrode leads were arranged in parallel without overlapping.

During testing, participants performed a unipedal stance on the pressure platform. Muscle signals were recorded for 13 s under two conditions: baseline (no stimulation) and during plantar micro-vibration stimulation. Raw electromyographic (EMG) data were collected and processed using MATLAB to calculate root mean square (RMS) values.

SPSS software was used for statistical evaluation. Normality of RMS-EMG data distribution was assessed via Q-Q plots and Kolmogorov-Smirnov tests. Paired *t*-tests compared EMG metrics between control and stimulation conditions, with statistical significance set at *p* < 0.05.

### 2.7. Effectiveness of Micro-Vibration Stimulation in Enhancing Training Outcomes

Prior to testing, the vibratory insole was positioned on the instrumented pressure platform (FDM-T, Zebris Medical GmbH). Before each test session, all participants received the same standardized verbal instructions. During the static standing task, they were instructed to maintain balance as much as possible. During the dynamic leaning task, they were instructed to reach the maximal leaning angle as much as possible within a safe range. Following palpation of four extrinsic foot muscle bundles—medial gastrocnemius (MG), tibialis anterior (TA), peroneus longus (PL), and extensor digitorum longus (EDL)—eight pre-gelled surface-adhesive bipolar Ag/AgCl electrodes (Shenfeng Medical Devices, Shanghai, China) were affixed to the skin surface.

Each participant was tested sequentially under three conditions: a control phase wearing a standard HI-POLY insole without vibration output, an active vibration phase with the micro-vibration insole delivering continuous plantar white-noise stimulation, and a post-rest phase following a ten-minute interval after vibration cessation. The identical testing protocol was performed in each phase. It began with a 13 s static unipedal stance on the dominant limb to record baseline postural stability. This was immediately followed by a multidirectional maximal lean test, where participants performed controlled maximal body leans while maintaining single-leg support. They sequentially leaned anteriorly, posteriorly, leftward, and rightward, holding each endpoint posture for five seconds to challenge dynamic postural control limits. The mandatory ten-minute interval between conditions served to mitigate muscle fatigue, ensure data independence, and control for potential sensory after-effects.

Surface electromyography signals were filtered using a Hamming bandpass filter with a frequency range of 10 to 500 Hz. Muscle activation was quantified via frequency-domain analysis using the Fast Fourier Transform to yield power spectral density values. For the multidirectional lean test, these values for each muscle were normalized to the maximum value recorded within the same directional condition to standardize comparisons. Sway parameters, including CoP area, path length, and velocity, were recorded for each trial. The CoP area was specifically estimated by fitting a 95% confidence ellipse to the anterior-posterior and mediolateral coordinates. Raw EMG data were collected at a sampling frequency of 2000 Hz using a MegaWin ME6000 system from Finland. The signals were bandpass-filtered from 10 to 500 Hz and subjected to a 48 to 52 Hz notch filter to reduce powerline interference. Root mean square EMG values were subsequently calculated over the entire sixty-second trial duration using a custom script in MATLAB from The MathWorks, based in Natick, MA, USA.

Statistical analysis was performed using SPSS software from IBM, USA. The normality of the CoP and root mean square EMG data distributions was assessed via Q-Q plots and Kolmogorov-Smirnov tests. For the within-subject comparisons (Control vs. Vibration and Control vs. Rest), repeated measurements within each participant were first averaged within condition, and paired *t*-tests were then performed at the subject level (*n* = 8) for the Control vs. Vibration and Control vs. Rest comparisons. For the between-group comparisons involving Cohort 2 and Cohort 3, independent-samples *t*-tests were used because the two cohorts consisted of different participants. The significance level was set at *p* < 0.05. Furthermore, correlations between CoP area and lower-limb muscle activity were examined using statistical tools implemented in MATLAB.

## 3. Results

Six participants were recruited for this study, with each individual tested under threshold-level vibration conditions across seven distinct stimulation site configurations. Significant differences in CoP confidence ellipse areas were observed between configurations (Figure 1c). Four participants (Subjects 2–4 and 6) exhibited minimal CoP areas during full-combination stimulation (Configuration 7), indicating optimal balance performance. Subject 1 showed superior balance during isolated fifth metatarsal (M5) stimulation compared to full-combination. Subject 5 achieved lowest CoP area with M1 + M5 stimulation, with full-combination ranking second. Full-combination stimulation improved balance capacity by 59.17% relative to maximal CoP area baselines.

This study evaluated the effects of different combinations of foot vibration sites on the CoP (Center of Pressure) envelope area in young healthy subjects. The results demonstrated that full-site vibration exhibited the most effective reduction in CoP envelope area among the seven combinations tested. Notably, certain subjects exhibited higher variability in CoP envelope area across vibration conditions, with this variability influenced by body weight and height. Subject 1, who presented the lowest anthropometric measurements (height and weight) among the cohort, displayed the largest fluctuation magnitude in CoP parameters, with a maximum difference of 182 observed between the first-fifth metatarsal combination vibration and fifth metatarsal vibration conditions.

**Figure 5:**
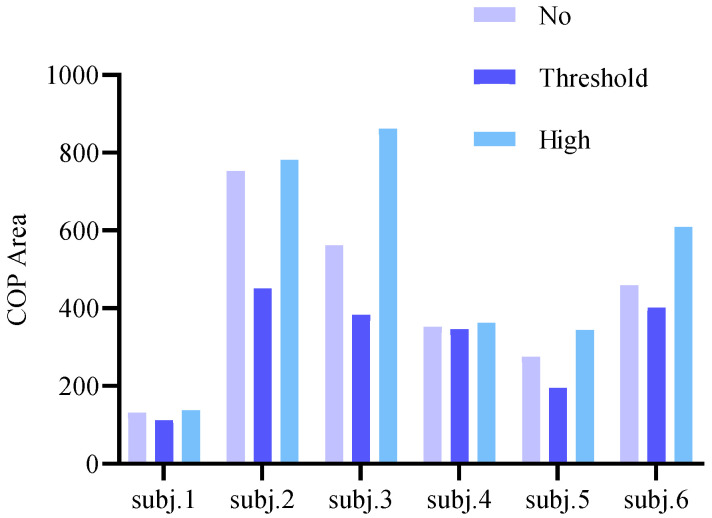
Effects of varying stimulation intensities on balance performance in subjects.

Regarding the minority of subjects (Subjects 1 and 5) whose optimal vibration combination did not correspond to full-site vibration (Figure 5), this phenomenon was attributed to their superior postural control capabilities. These individuals demonstrated lower amplitude variations in CoP envelope area, with their full-vibration condition values ranking second only to the minimum observed values. Consequently, the non-optimal status of full-vibration combinations for these particular subjects represents an expected outcome within the normal physiological response spectrum.

As shown in Table 1, none of the six participants exhibited significant changes (*p* > 0.05) in RMS-EMG values when comparing pre- and post-vibration conditions under threshold-level stimulation.

Summary statistics for sway parameters under the Control, Vibration, and Rest conditions are presented in Table 2. Analysis was conducted at the subject level after averaging repeated measurements within each condition for each participant. Compared to the control group, the white-noise vibration condition exhibited a 172.18 mm^2^ reduction in CoP envelope area, corresponding to a 39.32% decrease relative to control in overall balance performance. Statistical analysis revealed a significant reduction (*p* < 0.001) in mean CoP area in the white-noise vibration group relative to the control condition. Importantly, CoP area in the Rest condition also remained significantly lower than that in the Control condition, decreasing by about 19%, which suggests that part of the vibration effect was retained after cessation of stimulation. For directional displacement measures, the Rest condition showed significant increases in the medial and lateral directions compared with the Control condition, whereas the anterior and posterior directions showed only increasing trends without statistical significance. Overall, these findings indicate that plantar white-noise vibration produced an immediate balance-enhancing effect and that part of this effect was still preserved after the 10-min rest interval, although the magnitude of improvement was weaker than that observed during active vibration.

To clarify the distinct contributions of long-term multidirectional balance training and the specific effect of plantar vibration, we analyzed the longitudinal data from Cohort 3, the training control group, over the 8-week period and compared it with the outcomes from Cohort 2, the vibration intervention group.

The analysis confirmed that the training regimen itself was effective. Participants in Cohort 3, who completed 8 weeks of multidirectional balance training without any vibratory stimulation, demonstrated a significant improvement in static postural stability. Their CoP envelope area decreased from a pre-training average of 440.21 ± 189.32 mm^2^ to a post-training average of 380.47 ± 175.18 mm^2^, a statistically significant within-group improvement. This result confirms that the protocol induced a substantial “training effect,” capable of enhancing postural control in healthy young adults independently.

The critical comparison, however, lies between the two groups after the intervention. As detailed in Table 3, post-intervention between-group comparisons were conducted using independent-samples *t*-tests. The final postural stability achieved by Cohort 2, which received training combined with plantar vibration, was significantly superior to that of Cohort 3. The mean CoP area for Cohort 2 was 265.66 ± 188.60 mm^2^, which was markedly smaller than the 380.47 ± 175.18 mm^2^ observed in Cohort 3 (*p* = 0.006). Significant between-group differences were also observed for maximal anterior, posterior, medial, and lateral displacement measures.

Quantitatively, while systematic training alone conferred an approximately 13.6% improvement in balance (based on CoP area reduction), the integrated intervention of training with optimized plantar white-noise vibration yielded a 51.9% improvement. These findings suggest that plantar vibratory stimulation may contribute additional balance improvements beyond those attributable to balance training alone. Its effect was associated with greater improvement than that produced by training alone.

**Figure 6:**
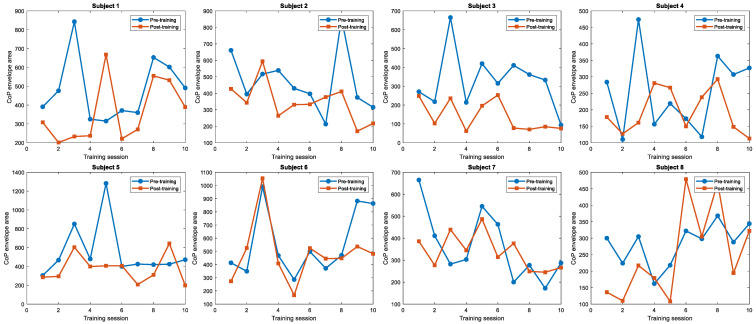
Changes in Observed Parameters Between the Control and Vibration Groups Across Eight Participants.

**Figure 7:**
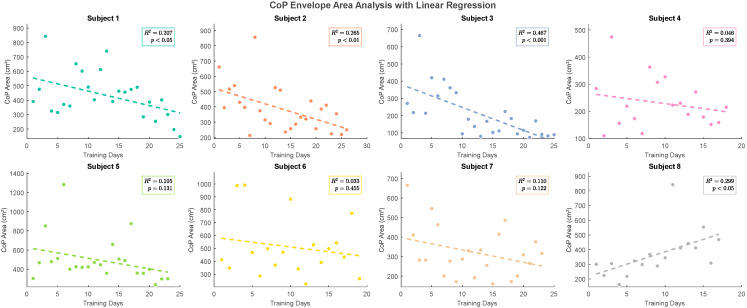
Changes in Subjects’ Observed Indicators Under Long-Term Stochastic Resonance Balance Training.

For the eight subjects, each underwent vibratory testing under threshold-intensity conditions. As illustrated in Figure 6, Longitudinal data analysis over the recent 10 trials revealed a consistent reduction in the center of pressure (CoP) envelope area in the vibration experimental group compared to the blank control group during each comparative session. As illustrated in Figure 7, linear regression analysis of nearly 20 training sessions revealed a declining trend in CoP envelope area for most subjects, with slope coefficients of −10.05, −10.14, −13.31, −3.747, −12.4, −1.993, −6.313 and 18.91, respectively. These results suggest improved balance performance across participants and support the potential efficacy of the white-noise-based plantar vibratory stimulation device. Notably, Subjects 1–4 and 7 exhibited the most pronounced improvements, while Subjects 5 and 6 showed minimal changes. Conversely, Subject 8 demonstrated an upward trend in CoP envelope area, reflecting diminished balance performance.

This study evaluated the short-term effects of white-noise-based plantar vibratory stimulation on CoP envelope area in young healthy subjects. The findings suggest that a majority of participants exhibited reduced CoP envelope area, indicating improved postural performance. However, Subjects 5 and 6 displayed limited efficacy, and some individuals showed no statistically significant reductions in CoP metrics. These variations may stem from multifactorial influences on balance control, including psychological states, transient muscle fatigue, and environmental interference.

These findings indicate that the response to long-term plantar white-noise vibration was heterogeneous across participants. Most participants exhibited a downward trend in CoP envelope area, whereas one participant showed an opposite trend.

## 4. Discussion

The data revealed a significant reduction in CoP confidence ellipse area under threshold-intensity vibration stimulation compared to the non-vibration condition (*p* < 0.05), indicating attenuated postural sway amplitude and enhanced balance performance. The threshold vibration group demonstrated a 21.65% mean improvement in balance capacity relative to the control cohort and a 33.05% increase compared to the high-intensity vibration group. In contrast, maximal-intensity vibration significantly expanded the CoP confidence ellipse area (*p* < 0.01), suggesting compromised postural stability, consistent with observations reported by Nicholas et al. [45]. These findings confirm the efficacy of white-noise vibration at threshold intensities for optimizing balance control mechanisms in young adults.

Notably, the present study observed that threshold-level plantar vibration reduced CoP area without altering muscle activation patterns. While the findings may be consistent with sensory-level modulation, the present study did not detect significant changes in ankle muscle activation under the current static testing condition. The observed changes in CoP area may be more appropriately interpreted as postural control modulation rather than direct motor activation, and further research is needed to confirm the role of plantar vibration in sensory processing. This dissociation suggests that stochastic resonance operates primarily at the sensory level rather than the motor level. The random vibration likely lowers the detection threshold of plantar mechanoreceptors, enabling the central nervous system to detect subthreshold postural sway signals that would otherwise go undetected. This enhanced sensory input allows the central nervous system to generate more precise corrective torque without increasing overall muscle activation. This interpretation aligns with the stochastic resonance model proposed by Collins et al. [20]. Body mass index (BMI) may also have contributed to the observed variability in CoP responses; however, the current sample size did not permit a formal BMI-stratified analysis. This issue should be addressed in future studies.

Compared with existing studies, Bovonsunthonchai et al. [46] applied 150 Hz vibration to the quadriceps and gastrocnemius muscles in healthy young adults and found no significant changes in CoP parameters. This discrepancy may relate to differences in vibration location and intensity selection: the present study employed threshold-intensity multi-site plantar stimulation, whereas Bovonsunthonchai et al. used single-site muscle stimulation. The systematic review by Xie et al. [47] further confirmed that variations in vibration frequency and amplitude can lead to opposite effects on balance control, with subthreshold Achilles tendon vibration reducing CoP displacement while perceptible Achilles tendon vibration significantly increasing it.

Notable individual differences in CoP responses were observed across vibration intensities. Anthropometric factors, particularly lower body height correlating with a lower center of gravity, contributed to stronger baseline postural control, resulting in smaller CoP variability under varying stimulation intensities [48]. To amplify significant differences in CoP metrics for such individuals, interventions like plantar cryotherapy to reduce tactile sensitivity [49] or balance testing on uneven surfaces may be employed [50].

The dose-response investigation of plantar nerve stimulation during static standing revealed no significant enhancement in activation levels of lower extremity prime movers (*p* > 0.05). However, during dynamic balance tasks, participants demonstrated reduced CoP confidence ellipse area (*p* < 0.05), indicating improved reactive postural adjustments under destabilizing conditions. These findings indicate that the balance-related effects of plantar vibration may differ across task conditions; however, the present data do not allow a direct mechanistic linkage between dynamic performance changes and muscle activation findings [51].

Inter-subject response heterogeneity was observed in the present study. Although most participants showed a reduction in CoP area over time, variability remained across individuals. Although most participants showed a reduction in CoP area over time, variability remained across individuals. This heterogeneity may reflect differences in baseline balance capacity, anthropometric characteristics, or other unmeasured factors. These findings underscore the need for larger samples and personalized stimulation protocols in future studies. Initial variability in outcomes was attributed to multifactorial influences on balance control, including psychological state, transient muscle fatigue, and environmental distractions. Eighty-five percent of participants demonstrated improved postural stability. Inter-subject response heterogeneity underscores the need for personalized stimulation protocols.

For rehabilitation professionals, the dose-response relationship established in this study, specifically threshold-intensity full-site stimulation, provides specific parameter guidelines for incorporating plantar vibration into balance rehabilitation protocols. The 8-week training regimen could be adapted for patients with mild balance impairments, particularly those for whom conventional balance training yields insufficient improvement. The absence of significant changes in muscle activation levels in this study suggests that this intervention does not exacerbate muscle fatigue, making it suitable for patients with limited exercise tolerance, such as those with early-stage multiple sclerosis or mild stroke.

Several limitations of the present study must be acknowledged. First, the small sample size across all cohorts limits the generalizability of the results. Second, the intervention cohort had a gender imbalance, with a higher number of male participants. Third, the participants did not have specific training in lower-limb balance exercises, and their habitual physical activity levels were low. As all participants were mainly graduate students engaged in sedentary academic work and had no formal balance-training background. Future studies should include a larger, more balanced sample of participants and account for participants’ habitual physical activity and training backgrounds to better interpret the effects of similar interventions and strengthen the external validity of the findings.

## 5. Conclusions

This study investigated the effects of plantar white-noise vibration stimulation on CoP oscillations and extrinsic foot muscle activation patterns in healthy adults under varying stimulation configurations. Our findings suggest that full-site stimulation may demonstrate enhanced balance improvement efficacy compared to alternative stimulation site combinations, while plantar vibratory stimulation was not observed to significantly alter ankle joint muscle activation levels. Furthermore, long-term intervention appears beneficial for improving balance control capacity in healthy young adults. However, the small sample size and gender imbalance across cohorts limit the external validity of these findings, and larger randomized trials with more balanced participant characteristics are needed.

## Figures and Tables

**Table 1:** Muscle Activation Dynamics.

Extrinsic Foot Muscles	Foot Sensory Conditions		
	RMS-EMG		
	Control	Vibration	*p*-value
MG	38.07 (4.64)	44.43 (18.73)	0.438
TA	86.95 (58.04)	80.15 (36.14)	0.813
PL	74.92 (28.39)	82.21 (52.15)	0.770
EDL	105.37 (76.05)	98.18 (66.22)	0.865

**Table 2:** The sway parameters of CoP Area, Anterior, Posterior, Medial and Lateral obtained under three different foot sensory conditions. Values are expressed as mean.

Sway Parameters	Foot Sensory Conditions			Control-Vibration *p*	Control-Rest *p*
	Control	Vibration	Rest		
CoP Area (mm^2^)	437.84 (190.95)	265.66 (188.6)	353.29 (174.14)	0.0001	0.030
Anterior (mm)	98.68 (13.74)	123.38 (16.3)	103.57 (17.25)	0.105	0.087
Posterior (mm)	103.34 (21.58)	123.35 (22.1)	111.78 (22.74)	0.083	0.077
Medial (mm)	18.69 (6.32)	23.36 (5.6)	20.69 (7)	0.061	0.039
Lateral (mm)	16.82 (4.23)	20.33 (4.17)	18.57 (5.1)	0.005	0.016

**Table 3:** Comparison of Balance Parameters Between Cohort 2 (Vibration + Training) and Cohort 3 (Training Only) After 8 Weeks of Intervention.

Sway Parameter	Cohort 2 (Vibration + Training)	Cohort 3 (Training Only)	Between-Group *p*-Value
CoP Area (mm^2^)	265.66 (188.6)	380.47 (175.18)	0.006
Anterior (mm)	123.38 (16.3)	112.45 (15.88)	0.041
Posterior (mm)	123.35 (22.1)	110.89 (20.74)	0.038
Medial (mm)	23.36 (5.6)	19.95 (5.21)	0.047
Lateral (mm)	20.33 (4.17)	17.62 (4.05)	0.049

## Data Availability

Data are available upon request from the corresponding authors.

## Abbreviations

The following abbreviations are used in this manuscript:

CoP	Center of Pressure
EMG	Electromyography
SR	Stochastic Resonance
MG	Medial Gastrocnemius
TA	Tibialis Anterior
PL	Peroneus Longus
EDL	Extensor Digitorum Longus
RMS	Root Mean Square
CNS	Central Nervous System
ACL	Anterior Cruciate Ligament
M1	First Metatarsal
M5	Fifth Metatarsal
C	Calcaneus

## References

[1] Hua A., Guillaume M., Rodrigues S.T., Barbieri F.A., Bonnet C.T. (2025). Benefits of swaying while standing to higher selective attention in goal-directed visual tasks. Hum. Mov. Sci..

[2] Lubetzky A.V., Cosetti M., Harel D., Scigliano K., Sherrod M., Wang Z., Roginska A., Kelly J. (2025). Frequency analyses of postural sway demonstrate the use of sounds for balance given vestibular loss. Gait Posture.

[3] Alkhamis B.A., Elrefaey B.H., Alahmari K.A., Koura G.M., Alfaya F.F., Reddy R.S. (2025). Cervical proprioception, postural control, and pain: Unraveling the interconnected challenges in rheumatoid arthritis. J. Orthop. Surg. Res..

[4] Castellanos-Cruz J.L., Gómez-Medina M.F., Tavakoli M., Pilarski P., Adams K.D. (2022). Preliminary testing of eye gaze interfaces for controlling a haptic system intended to support play in children with physical impairments: Attentive versus explicit interfaces. J. Rehabil. Assist. Technol. Eng..

[5] Moen U., Nilsen R.M., Knapstad M.K., Wilhelmsen K.T., Nordahl S.H.G., Goplen F.K., Meldrum D., Magnussen L.H. (2025). Musculoskeletal Pain as a Risk Factor for Poor Dizziness Outcomes: A Longitudinal Study among Patients with Persistent Vestibular Dizziness. Phys. Ther..

[6] Mishra S., Jain A., Sharma P., Khan G., Chhibber B. (2024). Effects of Lower Limb Proprioceptive Training on Balance and Trunk Control Among the Adult Stroke Population. Cureus.

[7] Kovaleva A.V., Birukova E.A., Kubryak O.V. (2018). Postural control ability and autonomic and central nervous system parameters in healthy volunteers. Int. J. Psychophysiol..

[8] Lemay J.F., Duclos C., Nadeau S., Gagnon D.H. (2015). Postural control during gait initiation and termination of adults with incomplete spinal cord injury. Hum. Mov. Sci..

[9] Kuo A.D. (1995). An optimal control model for analyzing human postural balance. IEEE Trans. Biomed. Eng..

[10] Winter D.A., Patla A.E., Frank J.S. (1990). Assessment of balance control in humans. Med. Prog. Technol..

[11] Xu D., Zhou H., Quan W., Ma X., Chon T.-E., Fernandez J., Gusztav F., Kovács A., Baker J.S., Gu Y. (2024). New insights optimize landing strategies to reduce lower limb injury risk. Cyborg Bionic Syst..

[12] Gruben K.G., Shiozawa K., Sugimoto-Dimitrova R., Hogan N. (2024). Human foot force suggests different balance control between younger and older adults. J. Neurophysiol..

[13] Viseux F.J.F., Billot M., Handrigan G., Simoneau M. (2024). Ankle torque variance is a better indicator of balance control performance than plantar perceptual sensitivity threshold. J. Appl. Physiol..

[14] Aboutorabi A., Arazpour M., Bahramizadeh M., Farahmand F., Fadayevatan R. (2017). Effect of vibration on postural control and gait of elderly subjects: A systematic review. Aging Clin. Exp. Res..

[15] Priplata A.A., Patritti B.L., Niemi J.B., Hughes R., Gravelle D.C., Lipsitz L.A., Veves A., Stein J., Bonato P., Collins J.J. (2006). Noise-enhanced balance control in patients with diabetes and patients with stroke. Ann. Neurol..

[16] Nozaki D., Collins J.J., Yamamoto Y. (1999). Mechanism of stochastic resonance enhancement in neuronal models driven by 1/f noise. Phys. Rev. E.

[17] Stephen D.G., Wilcox B.J., Niemi J.B., Franz J., Kerrigan D.C., D’Andrea S.E. (2012). Baseline-dependent effect of noise-enhanced insoles on gait variability in healthy elderly walkers. Gait Posture.

[18] Lipsitz L.A., Lough M., Niemi J., Travison T., Howlett H., Manor B. (2015). A shoe insole delivering subsensory vibratory noise improves balance and gait in healthy elderly people. Arch. Phys. Med. Rehabil..

[19] Sacco C.C., Gaffney E.M., Dean J.C. (2018). Effects of White Noise Achilles Tendon Vibration on Quiet Standing and Active Postural Positioning. J. Appl. Biomech..

[20] Collins J.J., Priplata A.A., Gravelle D.C., Niemi J., Harry J., Lipsitz L.A. (2003). Noise-enhanced human sensorimotor function. IEEE Eng. Med. Biol. Mag..

[21] Orland G., Brown S., Jude E., Bowling F.L., Boulton A.J.M., Reeves N.D. (2024). Acute Effects of Vibrating Insoles on Dynamic Balance and Gait Quality in Individuals with Diabetic Peripheral Neuropathy: A Randomized Crossover Study. Diabetes Care.

[22] Cham M.B., Mohseni-Bandpei M.A., Bahramizadeh M., Kalbasi S., Biglarian A. (2018). The effects of vibro-medical insole on sensation and plantar pressure distribution in diabetic patients with mild-to-moderate peripheral neuropathy. Clin. Biomech..

[23] Assländer L., Giboin L.S., Gruber M., Schniepp R., Wuehr M. (2021). No evidence for stochastic resonance effects on standing balance when applying noisy galvanic vestibular stimulation in young healthy adults. Sci. Rep..

[24] Rogan S., Hilfiker R., Schenk A., Vogler A., Taeymans J. (2014). Effects of whole-body vibration with stochastic resonance on balance in persons with balance disability and falls history—A systematic review. Res. Sports Med..

[25] Kennedy P.M., Inglis J.T. (2002). Distribution and behaviour of glabrous cutaneous receptors in the human foot sole. J. Physiol..

[26] Melvill Jones G., Kandel E.R., Schwartz J.H., Jessell T.M. (2000). Posture. Principles of Neural Science.

[27] Massion J. (1994). Postural control system. Curr. Opin. Neurobiol..

[28] Janin M., Dupui P. (2009). The effects of unilateral medial arch support stimulation on plantar pressure and center of pressure adjustment in young gymnasts. Neurosci. Lett..

[29] Morioka S. (2013). A Plantar Perceptual Learning Exercise to Improve the Standing Balance of Elderly People. J. Nov. Physiother..

[30] Hoch M.C., Russell D.M. (2016). Plantar cooling does not affect standing balance: A systematic review and meta-analysis. Gait Posture.

[31] Nakatani M., Sasaki H., Kurisu S., Yamaoka H., Matsuno S., Ogawa K., Yamasaki H., Wakasaki H., Furuta H., Nishi M. (2011). Numbness and paresthesia in bilateral toes and soles, and disproportional sweating restricted to face and trunk are suitable symptoms useful for the diagnosis of diabetic symmetric polyneuropathy. J. Diabetes Investig..

[32] Horak F.B. (2006). Postural orientation and equilibrium: What do we need to know about neural control of balance to prevent falls?. Age Ageing.

[33] André-Deshays C., Revel M. (1988). Rôle sensoriel de la plante du pied dans la perception du mouvement et le contrôle postural. Méd. Chir. Pied..

[34] Do M.C., Bussel B., Breniere Y. (1990). Influence of plantar cutaneous afferents on early compensatory reactions to forward fall. Exp. Brain Res..

[35] Galica A.M., Kang H.G., Priplata A.A., D’Andrea S.E., Starobinets O.V., Sorond F.A., Cupples L.A., Lipsitz L.A. (2009). Subsensory vibrations to the feet reduce gait variability in elderly fallers. Gait Posture.

[36] Chien J.H., Ambati V.N.P., Huang C.K., Mukherjee M. (2017). Tactile stimuli affect long-range correlations of stride interval and stride length differently during walking. Exp. Brain Res..

[37] Yamashita S., Igarashi K., Ogihara N. (2021). Reducing the foot trajectory variabilities during walking through vibratory stimulation of the plantar surface of the foot. Sci. Rep..

[38] Pathak P., Moon J., Roh S.G., Roh C., Shim Y., Ahn J. (2022). Application of vibration to the soles reduces minimum toe clearance variability during walking. PLoS ONE.

[39] Song H., Wang Z., Siu K.C., Chien J.H. (2022). Applying supra- or sub-threshold plantar vibrations increases the toe clearance while stepping over an obstacle. J. Mot. Behav..

[40] Peterka R.J. (2002). Sensorimotor integration in human postural control. J. Neurophysiol..

[41] Oshima T., Nakase J., Kitaoka K., Shima Y., Numata H., Takata Y., Tsuchiya H. (2018). Poor static balance is a risk factor for non-contact anterior cruciate ligament injury. Arch. Orthop. Trauma Surg..

[42] McGuine T.A., Greene J.J., Best T., Leverson G. (2000). Balance as a predictor of ankle injuries in high school basketball players. Clin. J. Sport Med..

[43] Dingenen B., Malfait B., Nijs S., Peers K.H.E., Vereecken S., Verschueren S.M.P., Janssens L., Staes F.F. (2016). Postural stability during single-leg stance: A preliminary evaluation of noncontact lower extremity injury risk. J. Orthop. Sports Phys. Ther..

[44] Hertel J., Gay M.R., Denegar C.R. (2002). Differences in postural control during single-leg stance among healthy individuals with different foot types. J. Athl. Train..

[45] Kreter N., Rogers C.L., Fino P.C. (2021). Anticipatory and reactive responses to underfoot perturbations during gait in healthy adults and individuals with a recent mild traumatic brain injury. Clin. Biomech..

[46] Bovonsunthonchai S., Hengsomboon P., Tangluang S., Anusri P., Chotikul P., Phiwmou W. (2018). The effect of sound and vibration on postural balance in healthy young adults. Walailak J. Sci. Technol..

[47] Xie H., Song H., Schmidt C., Chang W.P., Chien J.H. (2023). The effect of mechanical vibration-based stimulation on dynamic balance control and gait characteristics in healthy young and older adults: A systematic review of cross-sectional study. Gait Posture.

[48] Eom G., Kwon Y.R., Kim D.Y., Ko J., Kim J.W. (2022). The influence of height on test-retest reliability of postural balance measures in healthy young adults. J. Mech. Med. Biol..

[49] Kenville R., Clauß M., Arup A., Ragert P., Maudrich T. (2024). No Effect of Intermittent Palm or Sole Cooling on Acute Training Volume during Resistance Exercise in Physically Active Adults: A Summary of Protocols. Sports.

[50] Aminiaghdam S., Blickhan R., Muller R., Rode C. (2017). Posture alteration as a measure to accommodate uneven ground in able-bodied gait. PLoS ONE.

[51] Sajedifar M., Fakhari Z., Naghdi S., Ansari N.N., Honarpisheh R., Nakhostin-Ansari A. (2023). Comparison of the immediate effects of plantar vibration of both feet with the plantar vibration of the affected foot on balance in patients with stroke: Preliminary findings. J. Bodyw. Mov. Ther..

